# Knocking down GALNT6 promotes pyroptosis of pancreatic ductal adenocarcinoma cells through NF-κB/NLRP3/GSDMD and GSDME signaling pathway

**DOI:** 10.3389/fonc.2023.1097772

**Published:** 2023-02-28

**Authors:** Mengyang Ding, Jingyu Liu, Honghui Lv, Yanlin Zhu, Yumiao Chen, Hui Peng, Sairong Fan, Xiaoming Chen

**Affiliations:** ^1^ Key Laboratory of Laboratory Medicine, Ministry of Education, School of Laboratory Medicine and Life Sciences, Wenzhou Medical University, Wenzhou, China; ^2^ Wenzhou Key Laboratory of Cancer Pathogenesis and Translation, School of Laboratory Medicine and Life Sciences, Wenzhou Medical University, Wenzhou, China; ^3^ Institute of Glycobiological Engineering, School of Laboratory Medicine and Life Sciences, Wenzhou Medical University, Wenzhou, China

**Keywords:** pancreatic ductal adenocarcinoma, GALNT6, pyroptosis, NF-κB, GSDME

## Abstract

**Background:**

Pancreatic ductal adenocarcinoma (PDAC), the most prevalent type of pancreatic cancer, is a highly lethal malignancy with poor prognosis. Polypeptide N-acetylgalactosaminyltransferase-6 (GALNT6) is frequently overexpressed in PDAC. However, the role of GALNT6 in the PDAC remains unclear.

**Methods:**

The expression of GALNT6 in pancreatic cancer and normal tissues were analyzed by bioinformatic analyses and immunohistochemistry. CCK8 and colony formation were used to detect cell proliferation. Flow cytometry was applied to detect cell cycle.The pyroptosis was detected by scanning electron microscopy. The mRNA expression was detected by qRT-PCR. The protein expression and localization were detected by western blot and immunofluorescence assay. ELISA was used to detect the levels of inflammatory factors.

**Results:**

The expression of GALNT6 was associated with advanced tumor stage, and had an area under curve (AUC) value of 0.919 in pancreatic cancer based on the cancer genome atlas (TCGA) dataset. Knockdown of GALNT6 inhibited cell proliferation, migration, invasion and cell cycle arrest of PDAC cells. Meanwhile, knockdown of GALNT6 increased the expression levels of interleukin-1β (IL-1β), interleukin-6 (IL-6), tumor necrosis factor-α (TNF-α) and interleukin-18 (IL-18), the release of inflammasome and an increasing of Gasdermin D (GSDMD), N-terminal of GSDMD (GSDMD-N), Gasdermin E (GSDME) and N-terminal of GSDME (GSDME-N) in PDAC cells. GALNT6 suppressed the expression of NOD-like receptor thermal protein domain associated protein 3 (NLRP3) and GSDMD by glycosylation of NF-κB and inhibiting the nucleus localization of NF-κB. Additionally, GALNT6 promotes the degradation of GSDME by O-glycosylation.

**Conclusion:**

We found that GALNT6 is highly expressed in pancreatic cancer and plays a carcinogenic role. The results suggested that GALNT6 regulates the pyroptosis of PDAC cells through NF-κB/NLRP3/GSDMD and GSDME signaling. Our study might provides novel insights into the roles of GALNT6 in PDAC progression.

## Introduction

Pancreatic cancer (PC), one of the most lethal cancers, is the seventh leading cause of cancer-related deaths worldwide, accounting for almost as many deaths (466,000) as cases (496,000) (1). Pancreatic ductal adenocarcinoma (PDAC) is the most common pancreatic neoplasm and accounts for more than 90% of all pancreatic tumors with highly aggressive and malignant, in which the average 5-year survival rates is less than 10% (2, 3). Most patients with pancreatic ductal adenocarcinoma are diagnosed at an advanced stage due to no specific symptoms and the lack of early diagnosis, which makes the treatment virtually impossible and the clinical cure rate is extremely low (4, 5). It is well known that pancreatic cancer is a disease of genetic alterations, such as mutations in the genes *KRAS*, *TP53*, *CDKN2A* (encoding p16) and *SMAD4* (6). These genomic alterations contribute to multifaceted defects in tumor suppressor mechanisms resulting in dysregulated growth signaling and inflammation, which are key aspects of PDAC (7). Therefore, there is an urgent need to exploit new molecular players and understand the underlying mechanism will be helpful to exploration of early diagnostic or treatment strategies for PDAC.

Glycosylation is a major post-translational modification of proteins and involved in almost all physiological and pathological processes, such as cell proliferation, adhesion, epithelial-mesenchymal transition, cellular signaling, and immune recognition (8). Cumulative evidence shows that aberrant glycosylation is the prevalently observed in tumor cells and cited as a hallmark of cancer (9). O-glycosylation, one of the major forms of glycosylation, plays a global influence on cancer development and progression, such as tumor cell dissociation and invasion, metastasis, tumour angiogenesis and immune surveillance (10, 11). Recently, studies showed that aberrant O-glycosylation is prevalent in pancreatic cancer, and highlight as positive regulator of tumorigenesis, tumor progression, therapeutic resistance and remodeling the tumor immune microenvironment (12). For instance, O-glycosylation of malate dehydrogenase 1 (MDH1) enhances the stability of the substrate-binding pocket and glutamine metabolism, which contributes to PDAC growth (13). GALNT6, an enzyme of the N-acetylgalactosyltransferase family that initiate O-linked glycosylation (O-GalNAcylation) *via* transferring GalNAc from UDP-GalNAc onto Ser/Thr residues of acceptor proteins has been reported to promote the progression of various tumors (14). Previous studies have reported that high GALNT6 expression was strongly linked to the development of the abnormal mucin O-glycosylation in the development of human ductal carcinoma *in situ* (15). However, the underlying mechanism of GALNT6 in PDAC is still unclear.

Pyroptosis, a novel type of programmed cell death, is featured by cell swelling and plasma membrane rupture, and mediated by the activation of a variety of caspases, especially caspase-1, which is activated by inflammasomes, leading to cleavage of gasdermin family proteins and releasing of pro-inflammatory cytokines, such as IL-1β and IL-18 (16, 17). During the past decades, growing evidences showed that pyroptosis plays a suppression function of the proliferation, invasion and metastasis of tumors, evokes anti-tumor immune responses, which providing a great opportunity in cancer therapy (17, 18). Luteolin inhibits tumor growth by inducing pyroptosis *via* caspase-1/GSDMD signaling (19). Mammalian Ste20-like kinase 1 (MST1) inhibits the proliferation, migration, invasion and cell spheroid formation of PDAC by inducing pyroptosis (20). Pyroptosis could be induced by several distinct pathways, such as canonical inflammasome pathway and caspase-3 mediated pathway (17). The canonical inflammasomes recruit pro-caspase-1 through the inflammasome adaptor apoptosis-associated speck-like protein containing a CARD (ASC), leading to self-cleavage and activation of caspase-1, which cleaves pro-IL-1β, pro-IL-18 and GSDMD, resulting the formation of the pores in the plasma membrane and secretion of IL-1β/IL-18, generating cell swelling and osmotic lysis (16, 17).

In the present study, the role of GALNT6 in pancreatic ductal adenocarcinoma cells was investigated. GALNT6 was overexpressed in pancreatic cancer. Knockdown of GALNT6 significantly inhibited the proliferation, migration and invasion of PDCA cells. Knockdown of GALNT6 promotes the phosphorylation of NF-κB, which was translocated into the nucleus, leading to the expression of NLRP3 and GSDMD. Meanwhile, knockdown of GALNT6 increased the levels of GSDME and suppressed its degradation. These findings suggested that knockdown of GALNT6 promoted pryoptosis *via* NF-κB/NLRP3/GSDMD and GSDME signaling, which may contribute to the inhibiting of PDAC cell growth. The results of this study provide new insight into the roles of GALNT6 in tumor development and a potential treatment for PDAC.

## Materials and methods

### Bioinformatic analyses

The data of pancreatic cancer tissues (n = 178) and normal tissues (n = 171) and RNA-Seq expression data of GALNT6 were derived from the TCGA database (https://portal.gdc.cancer.gov/). We compared the expression of GALNT6 in pancreatic cancer and normal tissues with Wilcoxon rank sum test. The characteristics of patients about TNM stage were recorded in TCGA-GTEx database (https://portal.gdc.cancer.gov/). Receiver operating characteristic (ROC) curve was used to differentiate pancreatic cancer from adjacent normal tissues.

### Cell culture

Human pancreatic ductal cell (HPNE), pancreatic ductal adenocarcinoma cells (CFPAC-1, BXPC-3, Patu-8988t, MIA PaCa-2, PANC-1) were obtained from Chinese Academy of Medical Sciences (Beijing, China). The cells (HPNE, Patu-8988t, MIA PaCa-2 and PANC-1) were cultured in DMEM (C11995500BT, Gibco, NY, USA) containing 10% fetal bovine serum (FBS, Clark Bioscience, Virginia, USA). The cells (CFPAC-1 and BXPC-3) were cultured in RPMI 1640 (C11875500BT, Gibco, NY, USA) containing 10% FBS. All cells were cultured at 37°C with 5% CO_2_ incubator.

### Plasmids construction and lentivirus transfection

Interference plasmid GV248/EGFP/Puro-ShGALNT6 and the control plasmid GV248/EGFP/Puro-ShNC were designed and synthesized by Shanghai Genechem Co., LTD. The overexpression GALNT6 plasmid was purchased from Guangzhou GeneCopoeia Co., LTD. Lentivirus were prepared in HEK293T cells, according to the manufacturer’s instructions. Viral supernatant was collected at 48 h after transfection and used to infect the BXPC-3 and Patu-8988t cells. Stable pools were generated by puromycin (Sigma, St. Louis, USA) selection for following assays. The NF-κB knockdown cells (BXPC-3 and Patu-8988t) were prepared by using NF-κB specific siRNA (purchased from Shanghai Genechem Co., LTD.).

### CCK8 assay

Cells were seeded in 96-well plates at a density of 3×10^3^ cells/well and cultured in DMEM (or RPMI 1640) medium containing 10% FBS. After 24 h, 48 h and 72 h, respectively, 10 µl CCK8 reagent (CK04, Dojindo, Kumamoto, Japan) was added and cultured at 37°C for 2 h. The absorbance values of each sample were measured at 450 nm by an automatic plate reader (Thermo Scientific, Massachusetts, USA).

### Colony formation assay

Cells were seeded in 6-well plates at a density of 3×10^3^ cells/well and cultured in DMEM (or RPMI 1640) medium containing 10% FBS. After 14 days, cells were fixed with ethanol at room temperature for 20 minutes, followed stained with crystal violet for 30 minutes. After dried and photographed, cell clones were counted by using Image J software (Version 1.6.0, National Institutes of Health, Bethesda, Maryland, USA)

### Wound healing assay

Cells were seeded in 6-well plates and grown up to about 90% confluency. Subsequently, a 10 μl pipette tip was used to form two parallel straining lines. Samples were imaged under a microscope (Nikon, Tokyo, Japan) at 0, 24 and 48 hours, respectively. The closure ratio of wounds were measured by using Image J and calculated according to the formula: percentage of wound closure = (the initial area-follow-up area)/the initial area.

### Transwell assay

Corning Transwell inserts (8-μm pore size, 3422, Corning, NY, USA) were used to access cell migration and invasion. For migration assay, cells (3×10^4^ cells/well) were seeded into the upper chambers of the inserts and cultured in 200 µl serum-free medium, while the lower chambers were filled with 200 µl medium supplemented with 20% FBS. After cultured for 24 h, the chambers were wished with PBS. Then cells were fixed with 4% paraformaldehyde and stained with crystal violet. The chambers were photographed by microscope, and cell numbers were counted. For invasion assay, the procedure was similar to migration experiment with the following modification: the upper chambers were coated with BD matrigel and cells were cultured for 48 h.

### Cell cycle analysis

In brief, cells were collected, washed twice with PBS, and fixed in ethanol at 4°C overnight. Then, samples were centrifuged at 1000 rpm for 5 min, the supernatant was discarded, and wash twice with PBS. Cells were resuspended in the staining buffer (propidium iodide (PI): RNase = 9: 1) and incubated for 30-60 min. Before detected, 200 mesh nylon omentum was used to filter the cells to reduce the cell mass. Finally, the samples were analyzed by Flow Cytometer (BD, New Jersey, USA). The proportion of G0/G1 phase was calculated.

### Cell apoptosis analysis

Annexin V-APC and phycoerythrin (PE) double staining were used for apoptosis detection. According to the manufacturer’s protocol (KGA1026, Lianke Biological Co., LTD, Hangzhou, China), 1×10^6^ cells were harvested, washed with PBS and stained with 10 µl Annexin V-APC and 5 μl PE for 30 min in a dark room. Before detected, 200 mesh nylon omentum was used to filter the cells to reduce the cell mass. Finally, the samples were analyzed by Flow Cytometer (BD, New Jersey, USA).

### Western blot

Western blot was utilized to detect the protein expression level in cells. Then cells were lysed with radio immunoprecipitation assay (RIPA, P0013B, Beyotime, Shanghai, China) lysate and 1% phenylmet- hanesulfonyl fluoride (PMSF, ST506, Beyotime, Shanghai, China). The supernatant was collected by centrifugation and the protein concentration was detected by bicinchoninic acid (BCA, P0010, Beyotime, Shanghai, China) kit. The protein samples denatured at 100°C for 10 minutes, and separated on sodium dodecyl sulfate polyacrylamide gel electrophoresis (SDS-PAGE). Then, the protein was transferred to a polyvinylidene fluoride (PVDF, IPVH00010, 0.45 µm, Millipore, Massachusetts, USA) membrane. The membranes were blocked in 5% nonfat milk in Tris Buffered Saline (TBST) with 0.1% Tween-20, and incubated with the primary antibody at 4°C overnight, followed by incubated with the secondary antibody at room temperature for 1 h. The primary antibody used in this study as following: anti-GALNT6 (sc-100755, 1:1000, Santa Cruz Biotechnology, California, USA), anti-NF-κB (sc-109, Santa Cruz Biotechnology, California, USA), anti-p-NF-κB (S536) (AF2006, 1:1000, Affinity Biosciences, Shanghai, China), anti-NLRP3 (ab263899, 1:1000, Abcam, Cambridge, UK), anti-GSDMD (db3846, 1:1000, Diagbio, Hangzhou, China) and anti-GSDME (db3341, 1:1000, Diagbio, Hangzhou, China). Finally, the protein signal was visualized by enhanced chemiluminescence (ECL, P10100, NCM Biotech, Suzhou, China) reagent, and analyzed by using Image J software.

For pyrrolidinedithiocarbamate ammonium (PDTC) treatment assay, cells are treated with the PDTC (HY-18738, MedChemExpress, New Jersey, USA) for 24 h (10μg/ml) in 6-well plates, then the protein was extracted for WB experiment. PDTC is dissolved in water according to the reagent instructions.

For TNF-α treatment assay, cells are treated with the optimal doses of TNF-α (C008, novoprotein, Suzhou, China) for 12 h (1μg/ml) in 6-well plates, then the protein was extracted for WB experiment. TNF-α is dissolved in distilled water according to the reagent instructions.

### Cell fractionation assay

According to the instructions of the nuclear and cytoplasmic protein extraction kit (P0027, Beyotime, Shanghai, China). In brief, for every 20 μl of cell precipitate, 200 μl of cytoplasmic protein extraction reagent A was added with PMSF. Ice bath for 10-15 min. Then 10 μl cytoplasmic protein extraction reagent B was added and violently shook at the highest speed for 5 s and centrifuged at 4°C for 16000 g for 5 min. Immediately collected supernatant, which is the extracted cytoplasmic protein. For the precipitation, 50 μl nuclear protein extraction reagent with PMSF was added, and the cell precipitation was completely suspended and dispersed by intense shock. Then centrifuged at 4°C at 16000 g for 10 min. The supernatant was the extracted nuclear protein.

### Quantitative real-time PCR

Total RNA was extracted by RNA prep Pure Cell Kit (CS14010, Invitrogen, CA, USA), according to the manufacturing protocol. The cDNA was obtained by reverse transcription using the kit (R122-01, Vazyme, Nanjing, China), and quantified with SYBR^®^ Premix Ex TaqTM (Perfect Real Time) qPCR kit (Q711-02, Vazyme, Nanjing, China), and detected by fluorescence quantitative PCR instrument (CFX96) (Bio-Rad, California, USA). The primer sequences were used as follows: GALNT6: 5’-AGAGAAATCCTTCGGTGACATT-3’ (forward), 5'-AGACAAAGSGCCACAACTGATG-3' (reverse); (reverse); β-actin (used as internal normalization control): 5’-CACCATTGGCAATGAG CGGTTC-3’ (forward), 5’-AGGTCTTTGCGGATGTCCACGT-3’ (reverse).

### Immunofluorescence assay

Cells were fixed with 4% paraformaldehyde for 30 min at room temperature, permeabilized with 0.5% Triton for 20 minutes at room temperature, then washed three times with PBS. After blocked by 5% BSA for 30 minutes at room temperature, cells were incubated with primary antibody at 4°C for 12-14 hours, then incubated with fluorescent-dye conjugated secondary antibody for one hour at room temperature. Finally, cells were stained with 4’, 6-diamidino-2-phenylindole (DAPI, P0131, Beyotime, Shanghai, China) and the images were observed and photographed by fluorescence microscope.

### Enzyme-linked immunosorbent assay

According to the manufacturer’s instructions, enzyme-linked immunosorbent assay (ELISA) Kit (Lianke Biological Co., LTD, Hangzhou, China) was used to detect the levels of inflammatory factors (human IL-1β, IL-6, TNF-α, IL-18) in the culture medium of cell supernatant.

### Co-immunoprecipitation assay

Cells were lysed in the logarithmic growth phase by RIPA buffer (RIPA: PMSF: phosphatase inhibitor = 100: 1: 2), and protein was obtained. The samples were pre-cleared with Protein A+G agarose beads, incubated with corresponding primary antibody at 4°C overnight, and rabbit or mouse IgG was used as the negative control. Then, the pre-washed Protein A+G agarose beads were added and incubated at 4°C for 2 h. The protein-antibody complexes were collected, washed with PBS for 3 times. After denaturing in metal bath at 100°C for 10 min, immunoprecipitated proteins were collected and the agarose beads were removed. The samples were analyzed by western blot.

### VVA lectin pull-down assay

Cells were lysed by RIPA buffer (RIPA: PMSF: phosphatase inhibitor = 100: 1: 2). Cell lysates were incubated with VVA-conjugated beads (AL-1233, Vector Laboratories, L.A., USA) at 4°C and rotated overnight (14-16 h). After centrifugated, the precipitated protein was collected and analyzed by western blot.

### Scanning electron microscopy

The cells were cultured to the appropriate density on the crawling slices, and fixed with 2.5% glutaraldehyde solution at 4°C overnight in the refrigerator. Then dehydrated by soaking in 30%, 50%, 70%, 80% and 90% alcohol for 10 minutes. After soaking in tert-butanol for 15 minutes, samples were dried by hexamethyldisilazane (HMDS) chemical drying method. After coating, samples were photographed by scanning electron microscope.

### Immunohistochemistry

Pancreatic cancer tissue chips were bought from Xi’an Taibsbio Biotechnology Co., Ltd (DPA243a). Immunohistochemistry was used to detect the expression of GALNT6 in pancreatic cancer tissues and normal tissues. The tissue sections were dewaxed, dehydrated and antigen repaired, then blocked with endogenous peroxidase, following by 5% BSA for 30 min at room temperature, and incubated overnight at 4°C with a drop of appropriate primary antibody (GALNT6, ab151329, 1:500, Abcam, Cambridge, UK). The microarray was incubated with biocatalytic secondary antibody at 37°C for 1 h, and 3, 5-diaminobenzidine (DAB) peroxidase substrate kit (PV-6001, ZSGB−BIO, Beijing, China) was used as the chromogen.

### Statistical analysis

Student’s *t*-test was used for comparison between the two experimental groups. Dunnett’s multiple comparison test was used, when two groups could not be considered to be of equal variance. Data was expressed as the mean value ± S.D of at least three repeated experiments. The *P*-value of less than 0.05 was considered to be statistically significant. GraphPad Prism 8.0.2 statistical software was used for mapping and statistical analysis.

## Results

### GALNT6 is elevated in pancreatic ductal adenocarcinoma

**Figure 1:**
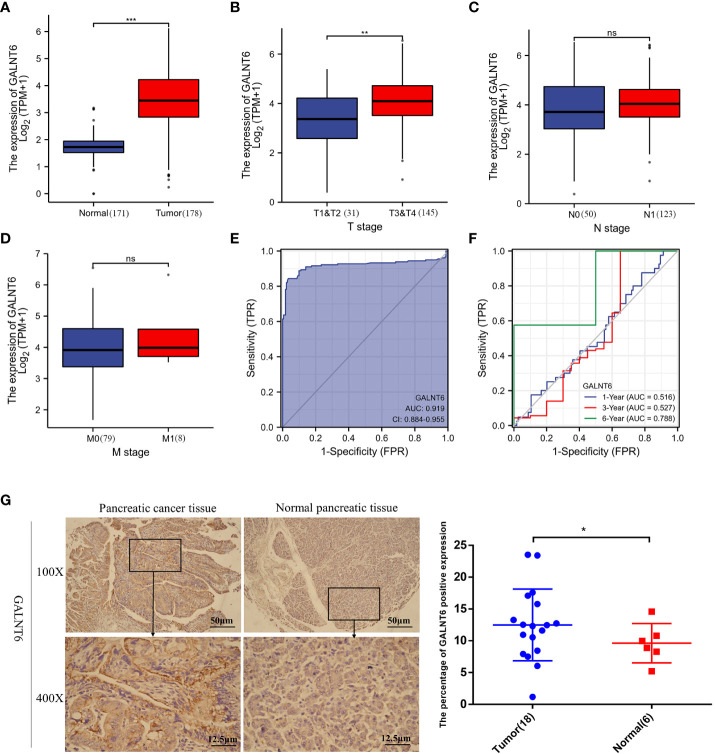
Analysis of GALNT6 in clinical database of pancreatic cancer patients. **(A–D)** Boxplots showed the expression of GALNT6 in pancreatic cancer and tumor TNM stages. **(E, F)** ROC and Time-dependent ROC curve showed more sensitive diagnosis of GALNT6 in pancreatic cancer (AUC=0.919). **(G)** Representative images of IHC. IHC was applied to detect the expression of GALNT6 in 18 pancreatic cancer tissues and 6 pancreatic normal tissues. ns, p >0.05; *p <0.05; **p <0.01; ***p <0.001.

To clarify the expression of GALNT6 in pancreatic cancer, the expression levels of GALNT6 were analyzed using TCGA database. As shown in **Figure 1A**, the level of GALNT6 in pancreatic cancer tissues (n = 178) was significantly higher than that in normal control (n =171). In addition, the association between the mRNA expression of GALNT6 and TMN stage was evaluated. GALNT6 was higher in T3/T4 stages than that in T1/T2 (**Figure 1B**). However, there was no significant difference between N0 and N1 (or M0 and M1) (**Figures 1C, D**). These results suggested that GALNT6 was associated with T stage, not the N or M stage. To investigate the diagnostic value of GALNT6 in pancreatic cancer, the ROC curve was analyzed. Results showed that GALNT6 had an AUC value of 0.919 (CI: 0.884-0.955) (**Figure 1E**). The time-dependent ROC results showed that the GALNT6 had a more sensitive diagnosis in six years than one or three years (**Figure 1F**). Furthermore, the expression of GALNT6 was detected in a tissue microarray of 18 pairs of pancreatic cancer tissues and normal control tissues by IHC. Results showed that the expression of GALNT6 in PDAC tissues was significantly higher than that in normal pancreatic tissues (**Figure 1G**). These findings suggested that the GALNT6 might be a positive regulator of PDAC.

### Down-regulation of GALNT6 suppresses the PDAC cells growth

**Figure 2:**
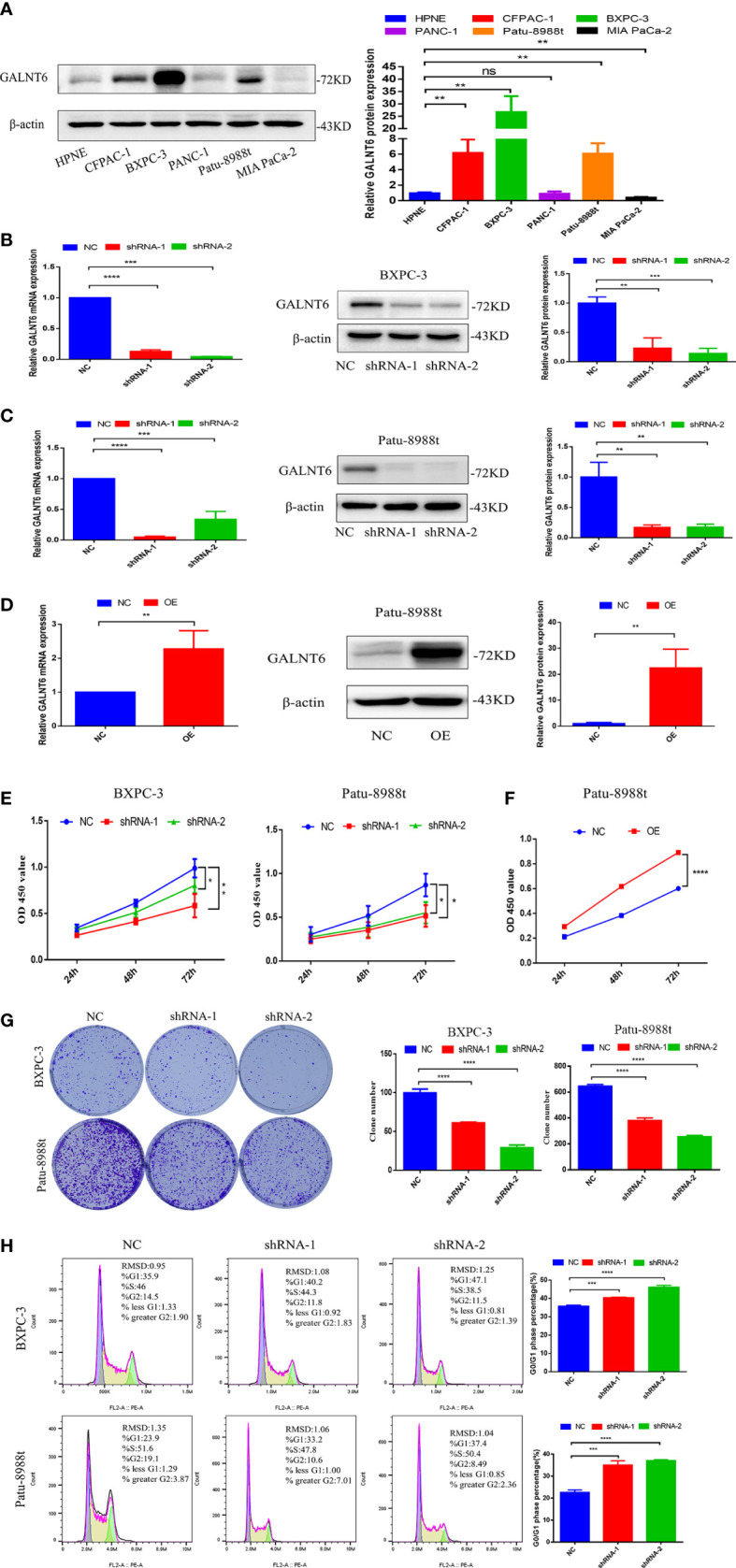
GALNT6 promotes the growth of PDAC cells. **(A)** Western blot was used to detect expression of GALNT6 in pancreatic cancer cell lines and pancreatic ductal cell. **(B, C)** The protein expression of GALNT6 was identified by western blot and the mRNA expression of shGALNT6 was identified by qRT-PCR. **(D)** The overexpression of GALNT6 in Patu-8988t cells was confirmed by western blot and qRT-PCR. **(E, F)** CCK8 was used to detect cell viability. **(G)** Plate cloning assay was used to detect cell proliferation. **(H)** Flow cytometry was applied to detect cell cycle. Each experiment was repeated at least three times independently, and the experimental data were expressed as mean standard deviation (X ± SD). ns, p >0.05; *p <0.05; **p <0.01; ***p <0.001; ****p <0.0001.

To validate the expression of GALNT6 in PDAC cell lines, CFPAC-1, BXPC-3, PANC-1, Patu-8988t, MIA PaCa-2 and pancreatic ductal cell (HPNE) were used. Results showed that the levels of GALNT6 in CFPAC-1, BXPC-3 and Patu-8988t were significantly higher than that in normal pancreatic cells (HPNE), and there was no difference between PANC-1 and HPNE cells (**Figure 2A**). To detect the effect of GALNT6 in PDAC progression *in vitro*, specific shRNA against GALNT6 was transferred into PDAC cell lines (BXPC-3 and Patu-8988t) to knockdown GALNT6 expression in these cells, and their corresponding scrambled vector (NC) was used as control. The expression of GALNT6 was significantly reduced in GALNT6 knockdown BXPC-3 cells (shRNA-1, shRNA-2), compared with NC cells (scrambled control) by western blot and qRT-PCR assay (**Figure 2B**). The similar effect was observed in the Patu-8988t cells (**Figure 2C**). Meanwhile, the overexpression of GALNT6 was constructed by transferring GALNT6 plasmids into Patu-8988t cells, and confirmed by western blot and qRT-PCR (**Figure 2D**). Knockdown of GALNT6 (shRNA-1, shRNA-2) significantly suppressed the growth of PDAC cells (BXPC-3, Patu-8988t) based on CCK8 assay (**Figure 2E**), and upregulated GALNT6 significantly increased the growth of Patu-8988t cells (**Figure 2F**). Similarly, the number of cell colonies was markedly decreased in shGALNT6 groups (shRNA-1, shRNA-2), compared with NC groups (**Figure 2G**). Cell cycle assay showed that knockdown of GALNT6 significantly increased the G1 phase distribution and decreased the G2 phase distribution, leading to increase the cell ratio in G0/G1 phase (**Figure 2H**), indicated that PDAC cells with GALNT6 knockdown were arrested at G0/G1 phase. These results showed that knockdown of GALNT6 suppressed the growth of PDAC cells.

### GALNT6 promotes migration and invasion of PDAC cells

**Figure 3:**
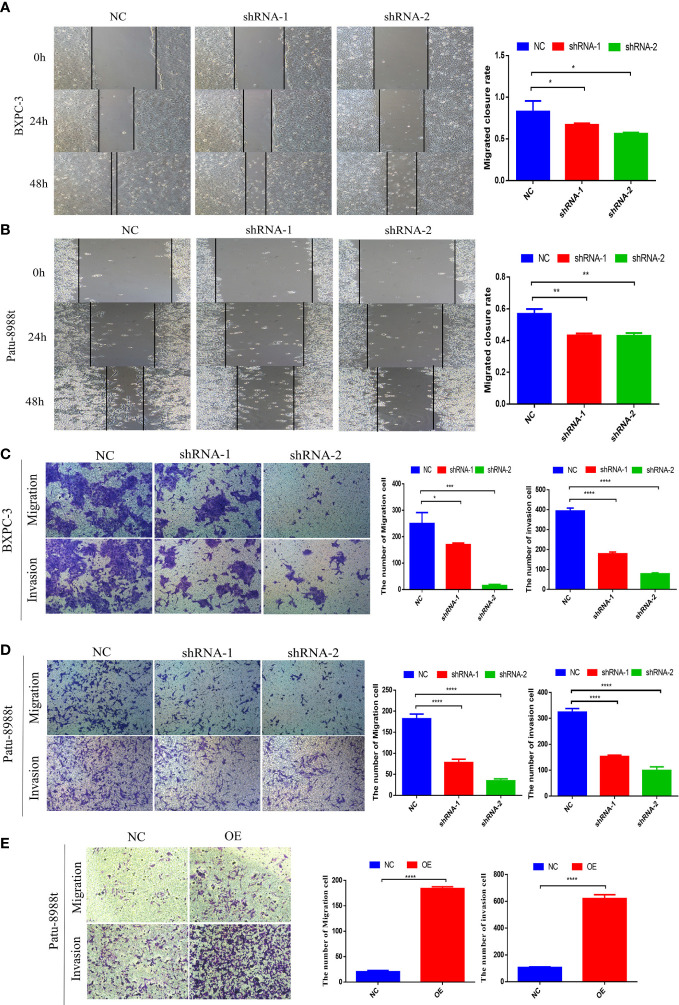
GALNT6 promotes the biological functions of PDAC cells. **(A, B)** Cell migration was detected by wound healing assay. **(C, D)** Transwell was used to detect the migration and invasion of Patu-8988t and BXPC-3 cells of GALNT6 knockdown. **(E)** Transwell assay was used to detect the migration and invasion of Patu-8988t cells of overexpression GALNT6. Each experiment was repeated at least three times independently, and the experimental data were expressed as mean standard deviation (X ± SD). *p <0.05; **p <0.01; ***p <0.001; ****p <0.0001.

To evaluate the effect of GALNT6 on the migration and invasion of PDAC cells, wound healing and transwell assay were performed. Down-regulation of GALNT6 significantly inhibited the wound closure, compared with control cells (NC), suggesting that knockdown of GALNT6 inhibited the migration of PDAC cells (BXPC-3, Patu-8988t) (**Figures 3A, B**). Transwell assay showed that the number of BXPC-3 and Patu-8988t cells migrated through the membrane was significantly decreased in shGALNT6 groups (shRNA-1, shRNA-2), compared with corresponding NC group (**Figures 3C, D**). Consistent with results of wound healing assay, knockdown of GALNT6 inhibited the migration of PDAC cells. Matrigel invasion assay showed the invasion capacity of BXPC-3 and Patu-8988t cells was also significantly reduced in the shGALNT6 groups (shRNA-1, shRNA-2) (**Figures 3C, D**). As expected, the overexpression of GALNT6 significantly promoted the migration and invasion of Patu-8988t cells (**Figure 3E**). These results suggested that GALNT6 promotes the migration and invasion of PDAC cells.

### GALNT6 promotes inflammation and pyroptosis

**Figure 4:**
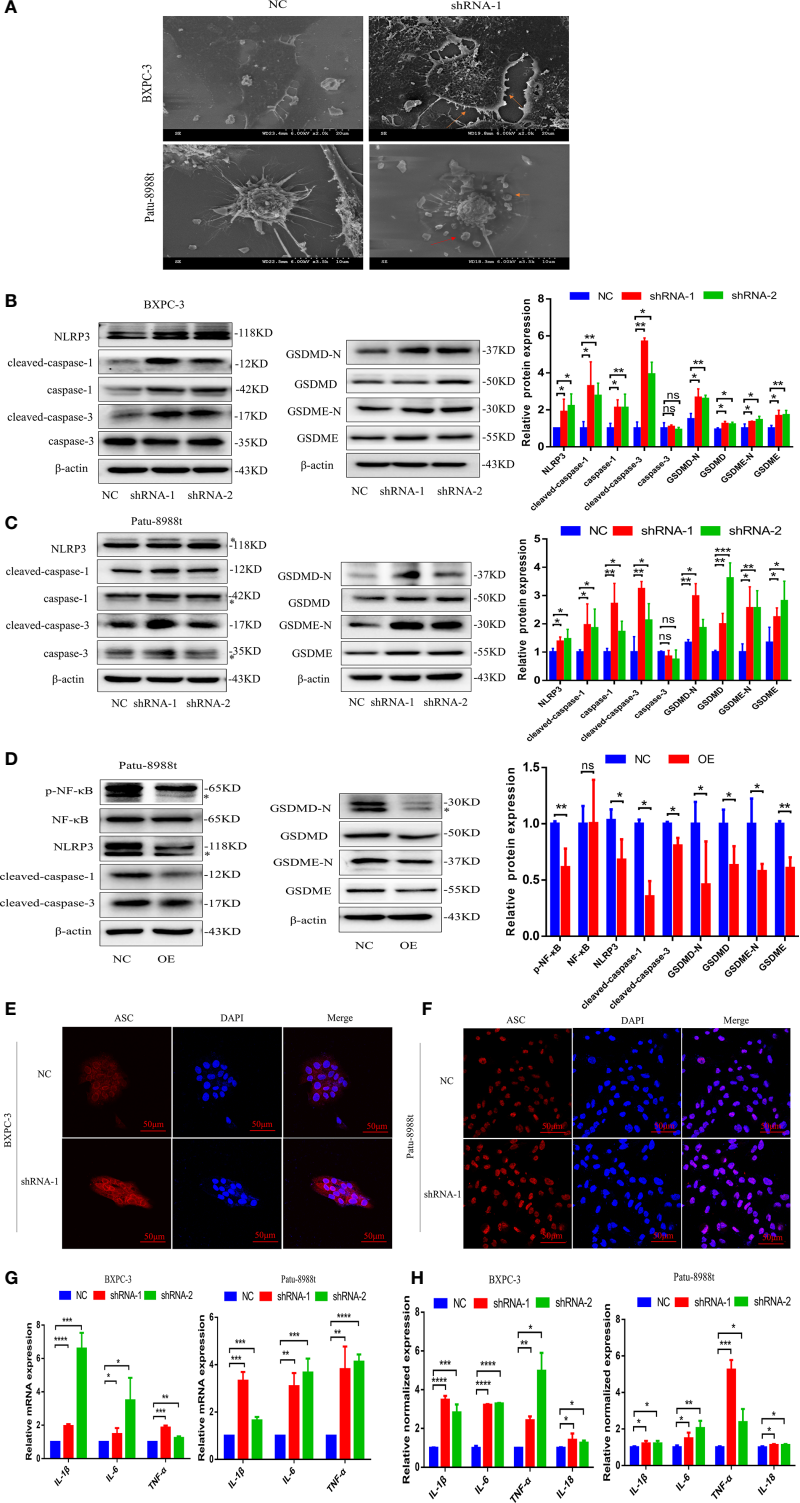
GALNT6 promotes the release of inflammatory factors and pyroptosis in PDAC cells. **(A)** Pyroptosis of BXPC-3 and Patu-8988t cells after knockdown of GALNT6 detected by SEM. Red arrows point to pyrosomes formed in cells and ruptured cell membranes. **(B, C)** Western blot assay was used to detect the changes of NLRP3, cleaved-caspase-1, caspase-1, cleaved-caspase-3, caspase-3, GSDMD-N, GSDMD, GSDME-N and GSDME expressions in BXPC-3 and Patu-8988t cells after knockdown of GALNT6. The asterisk indicates a heterozygous band. **(D)** The expression levels of p-NF-κB, NF-κB, NLRP3, cleaved-caspase-1, cleaved-caspase-3, GSDMD, GSDMD-N, GSDME and GSDME-N in overexpression GALNT6 Patu-8988t cells were measured by western blot assay. The asterisk indicates a heterozygous band. **(E, F)** ACS in BXPC-3 and Patu-8988t cells was analyzed by immunofluorescence. **(G)** The mRNA expression of IL-1β, IL-6 and TNF-α in BXPC-3 and Patu-8988t cells were detected by qRT-PCR. **(H)** ELISA was used to detect the levels of IL-1β, IL-6, TNF-α and IL-18 in BXPC-3 and Patu-8988t cells. Each experiment was repeated at least three times independently, and the experimental data were expressed as mean standard deviation (X ± SD). ns, p >0.05; *p <0.05; **p <0.01; ***p <0.001; ****p <0.0001.

Pyroptosis, as a new type of programmed cell necrosis, is mainly triggered by inflammasome and releases the intracellular inflammatory factors, and exerts tumor suppression function and evokes anti-tumor immunity responses (17, 21, 22). To further clarify the role of GALNT6 in PDAC, the effect of GALNT6 on PDAC pyroptosis was investigated. As shown in **Figure 4A**, bubble structure, cellular membrane ruptures and a number of vesicles were observed in GALNT6 knockdown group, indicating that knockdown of GALNT6 promoted the pyroptosis of PDAC cells. As shown in **Figure 4B**, the expression of NLRP3, caspase-1, cleaved-caspase-1, cleaved-caspase-3, GSDMD, GSDMD-N,, GSDME and GSDME-N were significantly increased in shGALNT6 groups (shRNA-1, shRNA-2), compared with that in NC group. Similar results were obtained in GALNT6 knockdown Patu-8988t cells (**Figure 4C**). The expression of caspase-3 was not affected by the GALNT6 knockdown in PDAC cells (**Figures 4B, C**). The expression of NLRP3, cleaved-caspase-1, cleaved-caspase-3, GSDMD, GSDMD-N, GSDME and GSDME-N was significantly reduced in GALNT6 overexpression Patu-8988t cells (**Figure 4D**). Inflammasomes typically contain ASC, caspase proteasomes, and a NOD-like receptor(NLR) family protein (such as NLRP3) or HIN200 family protein (such as AIM2). To further confirm the effect of GALNT6 on the inflammasome, ASC was examined by confocal laser scanning microscope. Results showed that knockdown of GALNT6 significantly increased the expression of ASC in PDAC cells (BXPC-3, Patu-8988t) (**Figures 4E, F**), suggesting that knockdown of GALNT6 increased the inflammasomes in PDAC cells. Activated inflammasome can promote the release of the pro-inflammatory cytokines, including IL-1β and IL-18. Results showed that the mRNA levels of IL-1β, IL-6 and TNF-α were markedly increased in GALNT6 knockdown PDAC cells (shRNA-1, shRNA-2), compared with that in corresponding NC group (**Figure 4G**). Meanwhile, ELISA assays results showed that the levels of IL-1β, IL-6, TNF-α and IL-18 were significantly elevated in BXPC-3 and Patu-8988t cells with GALNT6 knockdown (**Figure 4H**). These results suggested that GALNT6 knockdown may inhibit the growth of PDAC cell by inducing the pyroptosis of PDAC cells.

### Knockdown of GALNT6 promotes pyroptosis *via* NF-κB signaling in PDAC cells

**Figure 5:**
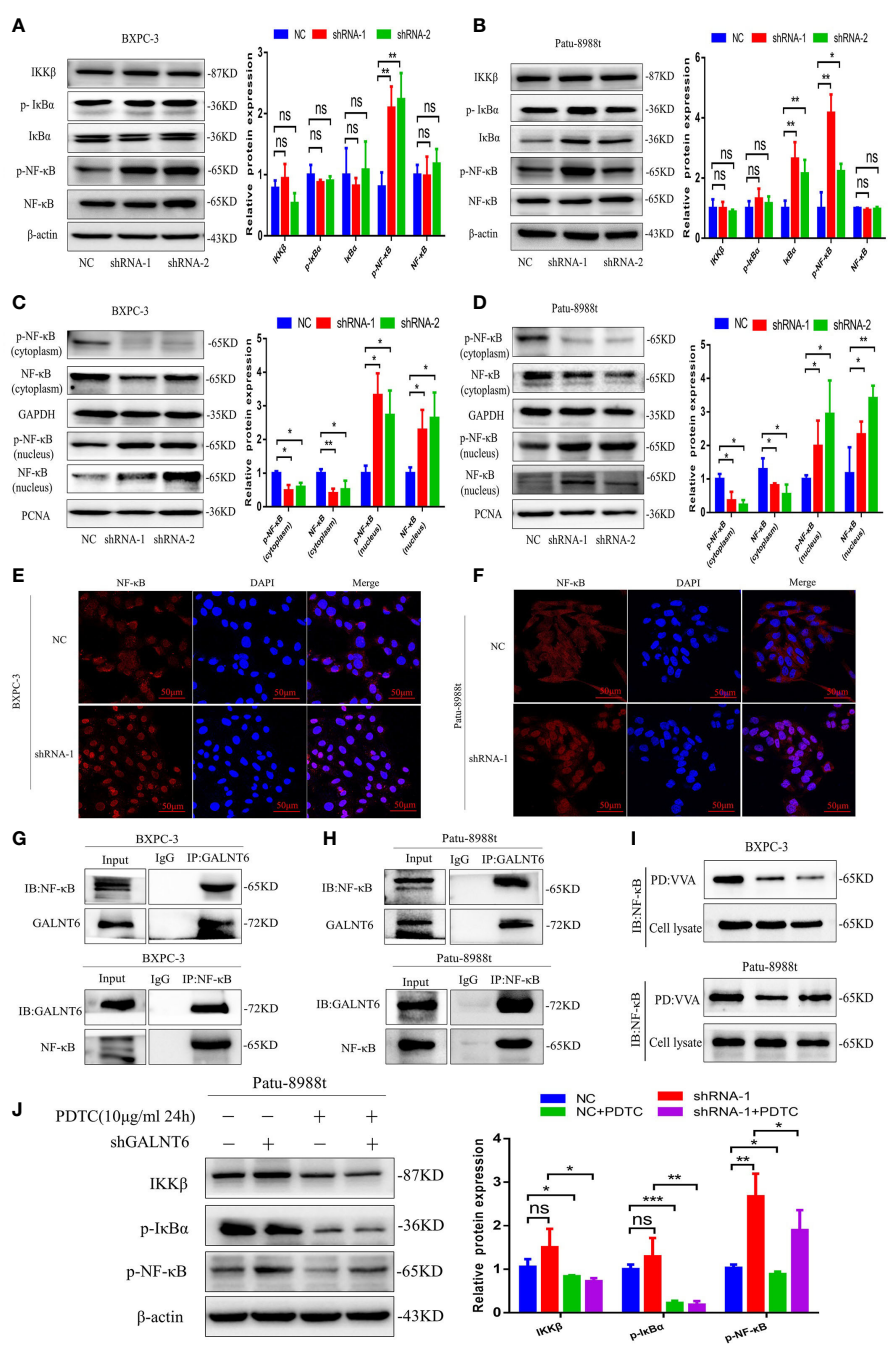
GALNT6 interacts with NF-κB. **(A, B)** Western blot was used to analyze the levels of IKKβ, p-IκBα, IκBα, p-NF-κB and NF-κB in BXPC-3 and Patu-8988t cells after knockdown of GALNT6. **(C, D)** Cell fractionation assay was used to detect the distribution of p-NF-κB and NF-κB in BXPC-3 and Patu-8988t cells after knockdown of GALNT6. **(E, F)** Immunofluorescence was used to detect the distribution of NF-κB in BXPC-3 and Patu-8988t cells after knockdown of GALNT6. **(G, H)** Co-IP assay was applied to determine the interaction of GALNT6 and NF-κB in BXPC-3 and Patu-8988t cells. **(I)** VVA pull-down assay was used to analysis glycosylation of NF-κB in BXPC-3 and Patu-8988t cells. **(J)** Western blot assay was used to detect the expression of IKKβ, p-IκBα, p-NF-κB in Patu-8988t cells after GALNT6 knockdown or PDTC treatment. Each experiment was repeated at least three times independently, and the experimental data were expressed as mean standard deviation (X ± SD). ns, p >0.05; *p <0.05; **p <0.01; ***p <0.001.

To elucidate the mechanism of GALNT6 in pryoptosis, the NF-κB signaling pathway, an important proinflammatory signaling pathway, which plays an important role in pyroptosis was investigated (23, 24). Results showed that the levels of NF-κB was unaltered by GALNT6 knockdown in PDAC cells (BXPC-3, Patu-8988t), however, the phosphorylation of NF-κB in PDAC cells was significantly increased by GALNT6 knockdown (shRNA-1, shRNA-2) (**Figures 5A, B**). The expression of p-NF-κB was significantly decreased in overexpressed GALNT6 cells (**Figure 4D**). There was no significant difference between the level of IKKβ and p-IκBα in GALNT6 knockdown groups and NC group (**Figures 5A, B**). To demonstrate the effect of GALNT6 on NF-κB signal, cell fractionation assay was performed to analyze the distribution of p-NF-κB and NF-κB, which is associated with the expression of proinflammatory cytokines (25). Results showed that knockdown of GALNT6 in PDAC cells significantly reduced the levels of p-NF-κB and NF-κB in cytoplasm and increased p-NF-κB and NF-κB accumulation in the nucleus, compared with control cells (NC) (**Figures 5C, D**). The distribution of NF-κB regulated by GALNT6 in PDAC cells were further confirmed by immunofluorescence (**Figures 5E, F**). Co-IP results showed that GALNT6 was interacted with NF-κB (**Figures 5G, H**). VVA pull-down results showed that knockdown of GALNT6 significantly reduced the glycosylation of NF-κB (**Figure 5I**). Furthermore, PDTC, an inhibitor of NF-κB (26), was used to investigate the effect of GALNT6 on NF-κB signaling. As shown in **Figure 5J**, PDTC significantly inhibited the expression of IKKβ and phosphorylation of IκBα with or without GALNT6 knockdown in Patu-8988t cells. However, the phosphorylation level of NF-κB reduced by PDTC was significantly increased by GALNT6 knockdown in Patu-8988t cells. These findings suggested that GALNT6 might regulate NF-κB signaling by glycosylation of NF-κB, which regulated the phosphorylation and distribution of NF-κB.

**Figure 6:**
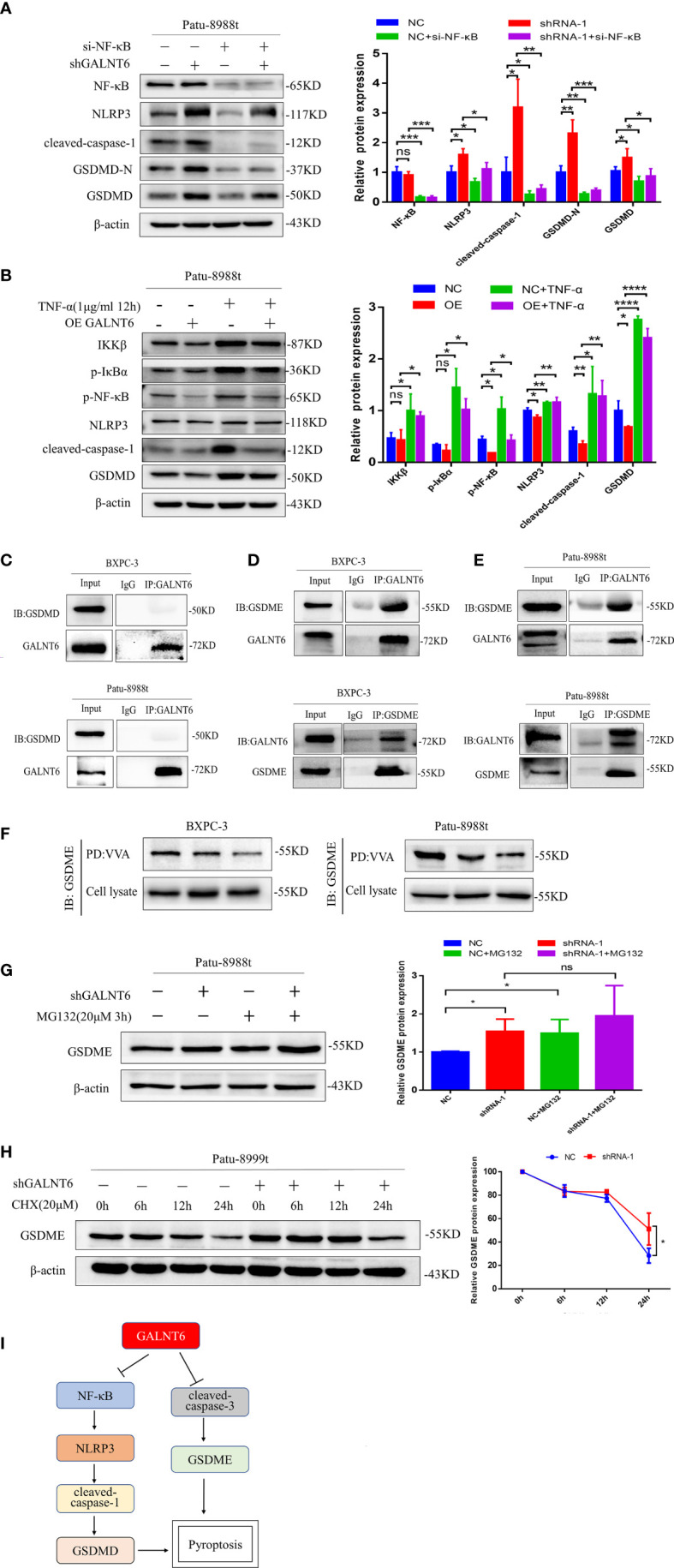
GALNT6 interacts with GSDME. **(A)** Western blot assay was used to detect the expression of NF-κB, NLRP3, cleaved-caspase-1, GSDMD-N and GSDMD in Patu-8988t cells after knockdown of NF-κB. **(B)** Western blot assay was used to detect the expression of IKKβ, p-IκBα, p-NF-κB, NLRP3, cleaved-caspase-1 and GSDMD in TNF-α treated Patu-8988t cells. **(C)** Co-IP assay was used to detect the interaction between GALNT6 and GSDMD in BXPC-3 and Patu-8988t cells. **(D, E)** Co-IP assay was used to detect interaction between GALNT6 and GSDME in BXPC-3 and Patu-8988t cells. **(F)** VVA pull-down assay was used to detect glycosylation of GSDME in BXPC-3 and Patu-8988t cells. **(G)** Western blot assay was used to detect the expression of GSDME in Patu-8988t cells after MG132 treatment. **(H)** Western blot assay was used to detect the expression of GSDME in Patu-8988t cells after CHX treatment. **(I)** Pattern signal diagram of GALNT6 regulates the pyroptosis of PDAC cells *via* NF-κB and GSDME. Each experiment was repeated at least three times independently, and the experimental data were expressed as mean standard deviation (X ± SD). ns, p >0.05; *p <0.05; **p <0.01; ***p <0.001; ****p <0.0001.

In order to further confirm the NF-κB regulated by GALNT6 is responsible for the pryoptosis in PDAC cells, siRNA against NF-κB was used to silence the expression of NF-κB. As shown in **Figure 6A**, knockdown of NF-κB drastically reduced the expression of NF-κB with or without knockdown of GALNT6, compared with their corresponding control Patu-8988t cells. The expression of NLRP3, cleaved-caspase-1, GSDMD and GSDMD-N was inhibited by NF-κB knockdown with or without GALNT6 knockdown, compared with corresponding control Patu-8988t cells (**Figure 6A**). On the one hand, TNF-α can be activated as a downstream inflammatory factor of NF-κB signaling pathway (27). on the other hand, TNF-α is a well-known upstream activator of NF-κB signaling pathway (28). To confirm the pyroptosis of Patu-8988t cells regulated by NF-κB signaling, TNF-α was used to active the NF-κB signaling. Results showed that the expression of IKKβ, p-IκBα, p-NF-κB, NLRP3, cleaved-caspase-1, GSDMD and GSDMD-N was significantly increased by TNF-α treated in Patu-8988t cells with or without GALNT6 overexpression, compared with their corresponding control cells (**Figure 6B**), indicating that TNF-α promoted the pyroptosis of Patu-8988t cells. When treated with TNF-α, the expression of NLRP3, cleaved-caspase-1 and GSDMD inhibited by GALNT6 was significantly elevated (**Figure 6B**). These results suggested that GALNT6 may inhibit the expression of extracellular inflammatory factors and intracellular inflammasomes by inhibiting the phosphorylation and nuclear translocation of NF-κB, thereby inhibiting the pyroptosis of PDAC cells.

### Knockdown of GALNT6 decreases the glycosylation of GSDME

Pyroptosis is produced not only by caspase-1/GSDMD induced by inflammasomes, but also regulated by the other gasdermin proteins, such as caspase-3/GSDME (29). GSDME converts relatively slow noninflammatory apoptosis into more rapid inflammatory pyroptosis (30). Our results showed that knockdown of GALNT6 increased the expression of cleaved-caspase-3, GSDME and GSDME-N and overexpression of GALNT6 reduced the expression of cleaved-caspase-3, GSDME and GSDME-N in PDAC cells (**Figures 4B-D**). Co-IP results showed that GALNT6 was interacted with GSDME, not GSDMD (**Figures 6C-E**). VVA pull-down results showed that knockdown of GALNT6 significantly reduced the glycosylation of GSDME (**Figure 6F**). To ascertain how GALNT6 regulates the synthesis or degradation of GSDME, the CHX (Cycloheximide, protein synthesis inhibitor) and MG132 (Z-Leu-Leu-Leu-Al, proteasome inhibitor) were used. When cells were treated with MG132, the levels of GSDME were significantly increased in Patu-8988t cells with or without GALNT6 knockdown (**Figure 6G**). After treated with CHX, the levels of GSDME were decreased with or without GALNT6 knockdown as time passed in Patu-8988t cells. However, the levels of GSDME in GALNT6 knockdown cells were higher than that in control cells at corresponding time (**Figure 6H**), suggesting that knockdown of GALNT6 inhibits the degradation of GSDME.

## Discussion

Pancreatic ductal adenocarcinoma (PDAC) is one of the deadliest cancers with a notoriously poor prognosis (31). Although the survival benefits for PDAC have improved, the therapy and prognosis of PDAC remains unsatisfactory. Pancreatic cancer is related to its extensive and complex tumor microenvironment (32), and is considered to have an exceedingly immunosuppressive environment, with numerous constituents and pathways hindering influential pancreatic cancer-targeted immune responses (33). Therefore, it is essential to exploiting the key driver factors and underlying mechanisms of PDAC progression to improve diagnosis and therapeutic effect. Glycosylation is one of the most common post-modification, and aberrant glycosylation has been cited as hallmark of cancer. Studies showed that the aberrant glycosylation described in PDAC contributes to pro-tumorigenic signaling pathways, metastatic capability and therapeutic resistance (12). However, the roles of alter glycosylation remain poorly understood in the progression of PDAC. Reports showed that dysregulation of GALNT3 and GALNT6 promote the metastatic phenotypes pancreatic cancer (34, 35). Here, we confirmed that GALNT6 was overexpression in PDAC, associated with advanced tumor stage, and had an AUC value of 0.919 in pancreatic cancer based on TCGA dataset. However, GALNT6 wasn’t overexpression in all PDAC cell lines, which may be linked to different types of pancreatic ductal adenocarcinoma. In this study, the upregulated GALNT6 cell lines (BXPC-3 and Patu-8988t) were used to further investigate the role and the underlying mechanism of GLANT6 in PDAC. Results showed that knockdown of GALNT6 inhibited cell proliferation, migration and invasion of PDAC cells, and induced G0/G1 phase arrest of PDAC cells, which might eventually inhibit the PDAC progression.

The infinite proliferation of tumor cells requires continuous shaping of a suitable microenvironment outside the tumor (36). Cancer invasion and distant metastasis are attributed to inflammatory factors in the tumor microenvironment, macrophage-driven chronic low-grade inflammation is an important feature of cancer (37, 38). However, the activated inflammatory response can induce the pyroptosis, an inflammatory cell death, which has been shown to be a more effective immunotherapy approach with fewer side effects compared with conventional immunotherapy, providing a great opportunity in treating solid tumors (17, 39). Pyroptosis-aroused immunological responses could convert immunosuppressive “cold” tumor microenvironment (TME) to immunogenic “hot” TME, which not only inhibits primary pancreatic cancer growth but also attacks the distant tumor (39). Pancreatic cancers frequently develop resistance to chemotherapy-induced cell apoptosis during the treatment, that targeting pyroptosis can be an alternative cancer treatment strategy (40). SEM results showed that knockdown of GALNT6 promoted the pyroptosis of PDAC cells. Pyroptosis can be induced by caspase-3 (inflammasome independent manner) or caspase-1 (inflammasome dependent manner) (17). An inflammasome is a protein complex, including NLRs, and recruit ASC, leading to recruit pro-caspase-1 and activate caspase-1 through autocleavage (17). Here, we found that knockdown of GALNT6 significantly increased the expression of caspase-1, NLRP3 and ACS in PDAC cells, and the level of cleaved-caspase-1 was also significantly increased, suggesting that knockdown of GALNT6 promoted the formation of ASC focus and activation of caspase-1 in PDAC cells, which might induce the pyroptosis of PDAC cells. Activated inflammasome can promote the release of the pro-inflammatory cytokines, including IL-1β and IL-18, which activate the innate immune system and strengthen the inflammatory response (41). Our results showed that knockdown of GALNT6 significantly increased the expression of inflammatory cytokines IL-1β, IL-18, IL-6 and TNF-α in PDAC cells. In additionally, GSDMD, the executor of pyroptosis, can be cleave by activated caspase-1 to generate GSDMD-N, which perforates the cell membrane and releases the IL-1β, IL-18, and other pro-inflammatory cytokines into the extracellular matrix (42). Here, we found that knockdown of GALNT6 significantly increased the expression of GSDMD in PDAC cells. These findings suggested that the Knockdown of GALNT6 induced the pyroptosis *via* the NLRP3/caspase-1/GSDMD signaling pathway, leading to inhibit the growth of PDAC cells.

As is well known, NF-κB pathway is a typical inflammatory signaling pathway, which is also involved in regulating the pyroptosis (43, 44). The activation of NF-κB results in the phosphorylation of IκB and the nuclear translocation of NF-κB, which promotes the expression of inflammatory cytokines, including TNF-α, IL-1β, IL-6 and IL-18, and further promoting the inflammatory response (45). The activated pro-inflammatory cytokine of IL-1β and IL-18, and subsequent activation of caspase-1 in the inflammasome that in turn induces pyroptosis and release of the active inflammatory cytokines (46). Our results showed that the phosphorylation of NF-κB was increased but NF-κB levels were unaltered in GALNT6 knockdown PDAC cells. Knockdown of GALNT6 promoted the translocation of NF-κB into nucleus and decreased the glycosylation of NF-κB, however, the IKKβ, IκBα and p-IκBα levels were unaltered. These findings suggested that the activation of NF-κB was directly regulated by GALNT6 knockdown, and subsequently induces transcription of IL-1β and IL-18 and activate inflammasome, leading to pyroptosis.

Furthermore, GSDME, one of the members of the gasdermin family, also plays important role in pyroptosis. GSDME is activated and cleaved by active caspase-3 to form N-terminal of GSDME, which could activate the canonical inflammasome pathway, leading to release of IL-18 and IL-1β and mediate pyroptosis (17). Studies showed that chemotherapeutic drugs convert the GSDME induced cell apoptosis to pyroptosis (47). Here, we found that knockdown of GALNT6 did not affect the expression of caspase-3, however, the level of cleaved-caspase-3, GSDME and GSDME-N was significantly increased by GALNT6 knockdown in PDAC cells, suggesting that knockdown of GALNT6 activated caspase-3, which promoted the cleavage of GSDME and induced the pyroptosis of PDAC cells. Furthermore, Co-IP and VVA pull-down results showed that GALNT6 was interacted with GSDME, and the glycosylation of GSDME by GALNT6 might promote the degradation of GSDME. These results suggested that caspase-3/GSDME was also regulated by GALNT6, which may attribute to the pyroptosis of PDAC cells.

In summary, we found that GALNT6 was overexpressed in PDAC and promoted cell proliferation, migration and invasion in PDAC cells. Knockdown of GALNT6 arrested cell cycle at G0/G1 phase and induced the pyroptosis of PDAC cells. GALNT6 regulated the pyroptosis of PDAC cells through NF-κB/NLRP3/GSDMD and GSDME signaling (The mechanism diagram is shown in **Figure 6I**), suggesting that GALNT6 may serve as a novel tumor therapeutic target for PDAC and providing novel insights into the roles of GALNT6 in PDAC progression.

## Data availability statement

The raw data supporting the conclusions of this article will be made available by the authors, without undue reservation.

## Author contributions

All authors contributed to this article and approved the submitted version. MD and XC designed the study. MD, JL, HL, YZ, YC, and HP performed the experiments. MD and JL analyzed the data and prepared the manuscript. SF and XC edited and approved the manuscript.
